# Genetic assessment of pathogenic germline alterations in lysosomal genes among Asian patients with pancreatic ductal adenocarcinoma

**DOI:** 10.1186/s12967-023-04549-x

**Published:** 2023-10-17

**Authors:** Youngil Koh, Hyemin Kim, So Young Joo, Seulki Song, Young Hoon Choi, Hyung Rae Kim, Byul Moon, Jamin Byun, Junshik Hong, Dong-Yeop Shin, Solip Park, Kwang Hyuck Lee, Kyu Taek Lee, Jong Kyun Lee, Daechan Park, Se-Hoon Lee, Jin-Young Jang, Hyunsook Lee, Jung-Ae Kim, Sung-Soo Yoon, Joo Kyung Park

**Affiliations:** 1 Department of Internal Medicine, Seoul National University Hospital, Seoul, Republic of Korea; 2 Department of Medicine, Samsung Medical Center, Sungkyunkwan University School of Medicine, Seoul, Republic of Korea; 3 Department of Biological Sciences, Institute of Molecular Biology and Genetics, Seoul National University, Seoul, Republic of Korea; 4 Department of Internal Medicine, College of Medicine, The Catholic University of Korea, Seoul, Republic of Korea; 5 Aging Convergence Research Center, Korea Research Institute of Bioscience and Biotechnology, Daejeon, Republic of Korea; 6 Structural Biology Department, Centro Nacional de Investigaciones Oncológicas (CNIO), Madrid, Spain; 7 Department of Molecular Science and Technology, Department of Biological Sciences, Ajou University, Suwon, Republic of Korea; 8 Department of Hematology/Oncology, Samsung Medical Center, Sungkyunkwan University School of Medicine, Seoul, Republic of Korea; 9 Departments of Surgery, Seoul National University College of Medicine, Seoul, Republic of Korea; 10 Department of Functional Genomics, KRIBB School of Bioscience, University of Science and Technology, Daejeon, Republic of Korea; 11 Cancer Research Institute, Seoul National University School of Medicine, Seoul, Republic of Korea; 12 Department of Health Sciences and Technology, SAIHST, Sungkyunkwan University, Seoul, Republic of Korea

**Keywords:** Pancreatic ductal adenocarcinoma, Lysosomal dysfunction, Autophagy, Germline variants, Genetic sequencing

## Abstract

**Background:**

Lysosomes are closely linked to autophagic activity, which plays a vital role in pancreatic ductal adenocarcinoma (PDAC) biology. The survival of PDAC patients is still poor, and the identification of novel genetic factors for prognosis and treatment is highly required to prevent PDAC-related deaths. This study investigated the germline variants related to lysosomal dysfunction in patients with PDAC and to analyze whether they contribute to the development of PDAC.

**Methods:**

The germline putative pathogenic variants (PPV) in genes involved in lysosomal storage disease (LSD) was compared between patients with PDAC (n = 418) and healthy controls (n = 845) using targeted panel and whole-exome sequencing. Furthermore, pancreatic organoids from wild-type and *Kras*^*G12D*^ mice were used to evaluate the effect of lysosomal dysfunction on PDAC development. RNA sequencing (RNA-seq) analysis was performed with established PDAC patient-derived organoids (PDOs) according to the PPV status.

**Results:**

The PPV in LSD-related genes was higher in patients with PDAC than in healthy controls (8.13 vs. 4.26%, Log_2_ OR = 1.65, P = 3.08 × 10^–3^). The PPV carriers of LSD-related genes with PDAC were significantly younger than the non-carriers (mean age 61.5 vs. 65.3 years, P = 0.031). We further studied a variant of the lysosomal enzyme, galactosylceramidase (GALC), which was the most frequently detected LSD variant in our cohort. Autophagolysosomal activity was hampered when GALC was downregulated, which was accompanied by paradoxically elevated autophagic flux. Furthermore, the number of proliferating Ki-67^+^ cells increased significantly in pancreatic organoids derived from *Galc* knockout *Kras*^*G12D*^ mice. Moreover, GALC PPV carriers tended to show drug resistance in both PDAC cell line and PDAC PDO, and RNA-seq analysis revealed that various metabolism and gene repair pathways were upregulated in PDAC PDOs harboring a GALC variant.

**Conclusions:**

Genetically defined lysosomal dysfunction is frequently observed in patients with young-onset PDAC. This might contribute to PDAC development by altering metabolism and impairing autophagolysosomal activity, which could be potentially implicated in therapeutic applications for PDAC.

**Supplementary Information:**

The online version contains supplementary material available at 10.1186/s12967-023-04549-x.

## Background

Despite medical advances, pancreatic cancer remains one of most fatal malignancies worldwide. According to Global Cancer Statistics 2020, pancreatic cancer, the seventh leading cause of cancer deaths worldwide, accounts for nearly as many deaths (466,000) as there are cases (496,000) because of its poor prognosis [1]. In most cases, delayed diagnosis is responsible for this dismal outcome: only 10–20% patients with pancreatic cancer are diagnosed with resectable disease [2]. Accordingly, early identification of high-risk populations and improvement in survival percentage are necessary. Approximately 10% of all pancreatic cancers are attributable to inherited risk factors [3, 4]. The genetic basis of familial or hereditary pancreatic cancer can be explained in 21% families based on previously described hereditary cancer-related genes and in 35% families based on low-frequency variants in other DNA repair genes [5]. More specifically, germline mutations in genes related to DNA instability, such as *CDKN2A, TP53, MLH1, BRCA2, ATM*, and *BRCA1,* are well known to be associated with the development of pancreatic adenocarcinoma (PDAC) [6].

Deleterious germline mutations are also evident in roughly 3.9–7% of pancreatic cancer cases lacking definitive family history [7, 8]. Consequently, knowledge regarding germline variant-driven susceptibility will be essential for understanding PDAC, as it will help in defining a high-risk population in cases of both familial and sporadic pancreatic cancer and will act as a rational background for novel anti-cancer drug development. Extensive research is warranted to elucidate the genetic mechanisms underlying PDAC. In addition to the aforementioned cancer-predisposing genes (CPGs), lysosomal storage diseases (LSDs) comprise more than 50 disorders caused by mutations in genes involved in the functioning of endosome–lysosome proteins [9]. Lysosomes are the main digestive compartments within cells and are closely linked to autophagy, a primary intracellular degradation system that derives its degradative abilities from lysosomes [10]. In cancer cells, lysosomes affect growth factor signaling via the endocytic degradation of growth factors, their receptors, or signal transduction mediators to modulate signaling output [11]. In addition, defective autophagy has been suggested to contribute to carcinogenesis, possibly owing to the reduced removal of defective organelles or damaged cells [12]. Accordingly, dysfunction and cancer development may be closely related. Indeed, a previous study has shown a possible association between rare variants of LSD-related genes and cancer [13]; compared with the average population, pathogenic variants of LSD genes were significantly enriched in the cancer cohort. PDAC is strongly associated with germline mutations in several LSD genes, including *SGSH*, *MAN2B1*, and *IDUA*. These results are consistent with evidence from a mouse model suggesting that autophagy suppresses cancer initiation [14]. Recently, adult-onset chronic diseases have been shown to originate from the heterozygote background of LSD gene variants. A good example is the association between Parkinson's disease and mutant GBA (encoding beta-glucocerebrosidase) heterozygote carriers [15].

We hypothesized that some heterozygote carriers of LSD genes may develop PDAC owing to the suppression of lysosomal dysfunction. In addition, once established, cancer cells use autophagy to promote survival during nutrient stress and recycle cell components to support a transformed phenotype [16], which is highly dependent on enhanced lysosomal function to facilitate the degradation, clearance, and recycling of cellular material delivered by increased rates of vesicle trafficking via autophagy and micropinocytosis [17–19]. Hence, impaired autophagic activity may contribute to cancer initiation; however, its role in established cancer cells may differ, as autophagy plays a biphasic role in cancer initiation and progression [20].

Taken together, the present study aimed to investigate the oncogenic effect of LSD heterozygote carrier status on PDAC. In particular, we aimed to (1) evaluate the clinical significance of rare LSD gene variants on large-scale in patient with PDAC and in healthy control cohorts, (2) focus on an ethnically homogenous population such as Koreans, since rare variant analysis is primarily affected by ethnicity, (3) evaluate the functional consequences of LSD gene dysfunction using mouse pancreatic organoids and human PDAC cell lines, and (4) assess characteristics of PDAC harboring rare LSD variants to understand the features of LSD-related PDAC using patient-derived organoids (PDOs).

## Methods

### Study cohort

In total, 418 patients diagnosed with PDAC between November 2011 and August 2020 at the Samsung Medical Center (SMC; n = 222) and Seoul National University Hospital (SNUH; n = 196) were prospectively enrolled and followed up until the end of 2021. Clinical and laboratory data were collected from the electronic health records (EHR). A cancer-free normal control (CFNC) cohort of 845 healthy volunteers was prospectively constructed at the SNUH Healthcare Checkup Center. The age distribution of the PDAC patients ranged from 35 to 87 years old. The age of all healthy volunteers was > 50 years, without a history of cancer, as proven by EHR, thereby excluding young age onset PDAC patients. This study was conducted in accordance with the principles of the Declaration of Helsinki, and the study protocol was approved by the SMC Institutional Review Board (IRB) and SNUH IRB (Seoul, South Korea). Written informed consent was obtained from all patients and volunteers, and all specimens were collected according to the IRB regulations and approval (IRB No. 2018-12-065, 1705-031-852).

### DNA sequencing analysis

Genomic DNA collected from peripheral blood was extracted using a QIAamp DNA tissue kit (Qiagen, Hilden, Germany) according to the manufacturer's instructions. Targeted panel or whole exome sequencing (WES) was used to evaluate the status of LSD germline variants in the study cohorts. First, targeted panel sequencing data were generated to identify LSD germline variants in 493 CFNC individuals. Then, the data generation method was changed for additional samples to identify variants in all genes. DNA from 352 additional CFNC individuals and 418 PDAC patients were used to generate WES data using an Illumina NovaSeq 6000 sequencer (Illumina, San Diego, CA, USA). Library capturing was performed using a probe from Integrated DNA Technologies. For the LSD panel sequencing, the DNA of 493 subjects from the CFNC cohort was sequenced using an Illumina HiSeq 2500 platform. Libraries were constructed using the ACCEL-NGS 2S DNA library kit (Swift Biosciences, Ann Arbor, MI, USA), which included 42 LSD genes (Additional file 4: Table S1).

Our in-house variant calling pipeline followed the Genome Analysis Toolkit (GATK) best practices recommended by the Broad institute [21, 22]. The sequence reads were aligned to the human reference genome (hg19) using the Burrows-Wheeler Aligner-MEM v0.7.10 algorithm [23]. Conversion to Binary Alignment Map (BAM) was performed using Picard v1.130. and deposited in the Sequence Read Archive (SRA bioproject accession # PRJNA929903). For insertions and deletions (indel) realignment, duplicated fragment elimination and base quality score recalibration were performed using the Genome Analysis Tool Kit (GATK v3.8.1, Broad Institute) [22]. Using the CollectHsMetrics of Picard tool, single nucleotide variants (SNVs) and indels were detected using the HaplotypeCaller of GATK v3.8.1 which is preferred for assessing germline variant calling by using de novo assembly of reads. Variants in each sample's genomic variant calling format (VCF) were merged, and a joint calling approach was performed using GATK to empower the variant discovery. Finally, sequencing errors were filtered out by assessing variant quality score recalibration (VQSR) and GATK's statistical modeling approach for variant filtration. Low-quality variants were discarded if the total coverage was less than 10, and sequencing reads with suspected bias were considered if the variant allele frequencies (VAF) were less than 20%. Additionally, variants listed in the ENCODE/DUKE [24] and DAC blacklist [25] regions were discarded, and only variants in the ENCODE/CRG GEM mappability region (75 mers) [26] were extracted to filter out well-known variants from low mappability regions. To generate a consistent set of variant filtration and functional annotations, the compiled variants in the VCF file were annotated using ANNOVAR [27] to perform filter-based functional annotation and a Variant Effect Predictor (VEP) [28] was used to explicate the gene-based information of canonical transcripts. In addition, to ensure that only rare germline variants were identified, we performed variant filtration using allele frequency (AF) information from The Genome Aggregation Database (gnomAD) [29] (gnomad.exomes.r2.1.1). The protein truncation variants and clinically validated variants in the ClinVar database was extracted as putative pathogenic variants (PPVs): (1) Tier1 variants were defined as protein truncating variants (PTVs), which included splice donor and acceptor site, frameshift indel, stop gain, and lost variants, as well as non-benign or likely benign loci annotated with Clinvar [30]; (2) Genetic variants with well-known clinical risks (pathogenic, likely pathogenic, association, and risk factor) and related phenotypes are clearly defined in ClinVar as Tier 2.

Most LSDs are caused by variants in genes that regulate lysosomal enzymes, which tend to be inherited in an autosomal recessive pattern. Somatic alterations were checked following the ‘two-hit’ hypothesis, in which a pathogenic variant occurs in one allele and the ‘second hit’ occurs in the other allele, leading to increased cancer risk. Therefore, somatic copy number alterations (CNA) across 42 LSD genes were determined using the CNVkit (https://github.com/etal/cnvkit) by comparing the tumor organoid DNA BAM files to germline samples (matched normal pancreatic blood). The mean read depths for each target (interval) were computed and normalized against a single reference of pooled standard samples, and the B-allele frequency was calculated. The observed log2 copy number ratios of the region < − 0.4 were derived as copy number loss, and CAN segments were visualized using the copy number package.

### RNA sequencing analysis

For RNA sequencing (RNA-seq), total RNA was isolated using a RNeasy mini kit (Qiagen). TruSeq stranded mRNA (Illumina) was used to prepare the RNA-seq libraries. The 150 bp paired-end sequencing of these libraries was performed using a NovaSeq 6000 sequencing system (Illumina). The quality of the cDNA libraries was evaluated using an Agilent 2100 bioanalyzer (Agilent, Santa Clara, CA, USA). Quality was confirmed using the NGS QC Toolkit (version 2.3.3). RNA-seq reads of pancreatic tumor organoids were aligned to the human reference genome (hg19) using spliced transcript alignment to a reference (STAR) version 2.5.3 a [31] expression count was estimated using RNA-seq by Expectation Maximization (RSEM-1.3.0) [32] and normalized using the EdgeR TMM method before performing differential expression analysis. To investigate how variants in the LSD-related genes affected gene expression, we analyzed the differentially expressed genes (DEGs) in LSD carrier and non-carrier groups using the transcript expression level from the RNA-seq data. Differential expression between LSD gene variant carriers and non-carriers was estimated using gene-specific read counts and the R package platform DESeq2 [33]. The log2 scale variance stabilizing transformation was completed using DEGs. Gene set enrichment analyses (GSEA) which is a functional enrichment analysis to identify association with group of genes was performed along with LSD carriers and non-carriers using Java GSEA application version 3.0. Functionally meaningful pathways were explored using Kyoto Encyclopedia of Genes and Genomes (KEGG) [34] and Gene Ontology (GO) databases of The Molecular Signatures Database (MSigDB). [35].

### Cell lines

Rare pathogenic germline variants in 42 LSD genes were screened in PDAC cell lines from the Cancer Cell Line Encyclopedia database [36]. The PK59 cell line with a *GALC* mutation (rs138577661 and rs137854543) was maintained in Roswell Park Memorial Institute (RPMI)-1640 medium with 10% fetal bovine serum (FBS). HPAFII, PANC1, AsPC1, and Capan-1 cells without *GALC* variants were maintained in Eagle’s minimum essential medium supplemented with 10% FBS, Dulbecco′s modified Eagle′s medium (DMEM) supplemented with 10% FBS, RPMI-1640 medium supplemented with 10% FBS, and Iscove's modified Dulbecco's medium supplemented with 20% FBS, respectively. All media and FBS were purchased from Gibco (Billings, MT, USA).

### Analysis of GALC enzyme activity

Endogenous GALC activity was detected using a lysosomal GALC analysis kit (Marker Gene Technologies, Inc., Eugene, OR, USA), according to the manufacturer’s instructions. Briefly, 4 × 10^6^ cells were harvested using the provided buffer and lysed using 2 × 30 s sonication cycles in a Bioruptor™ Pico. First, the lipidic fluorogenic substrate was incubated with 50 µg protein for 2 h at 37 °C. The fluorescence signals at excitation/emission = 365/454 nm were detected using a fluorometer (SYNERGY/HTS; BioTek Instruments, Winooski, VT, USA). The fluorescence of PK59 cells was normalized to the relative GALC activity.

### Analysis of autophagic flux

To detect the LC3B signal, 3–4 × 10^5^ cells were seeded in 6-well plates. The next day, the cells were washed twice with pre-warmed phosphate buffered saline (PBS) before treatment with 100 μM chloroquine (CQ; Sigma-Aldrich, St. Louis, MO, USA) for 1 h. After protein extraction, LC3B (Sigma-Aldrich) and β-actin (Santa Cruz) were detected using immunoblotting. The chemiluminescent signals were detected and visualized using a LAS-3000 luminescent image analyzer (FUJIFILM, Tokyo, Japan). The autophagy flux unit (A.F.U. = [LC3B-II/LC3B-I]_CO(+)_/[LC3B-II/LC3B-I]_CO(-)_) was calculated by analyzing the band intensities of LC3B-I and LC3B-II from three independent experiments using the ImageJ software.

PK59 and HPAF-II cells stably expressing GFP-LC3 were transfected with retrovirus (pBABEpuroGFP-LC3; Addgene, Watertown, MA, USA). After treatment with CQ (100 μM) for 5 h, GFP signals from LC3 were analyzed using a fluorescence microscope (Inverted Microscope Eclipse Ti-S; Nikon, Tokyo, Japan). Five images were selected to manually count the number of total and GFP-LC3 positive cells.

### Mouse pancreatic organoid culture

The protocols for mouse experiments were approved by the Institutional Animal Care and Use of Committee (IACUC) of Seoul National University (SNU-150724-4). *Kras*^*G12D/*+^ (Jackson Laboratory, Bar Harbor, Maine, USA) mouse was crossed with Pdx1-Cre mouse (Jackson Laboratory) to obtain *Kras*^*G12D/*+^; Pdx1-Cre mouse (*Kras*^*G12D*^). Since an activating point mutation of the KRAS oncogene in codon 12 (exon 2) occurs in the majority of PDAC cases (70–95%), the transgenic *Kras*^*G12D*^ mouse model is used in all pathophysiological studies [37]. Pancreatic ducts were isolated from wild-type and *Kras*^*G12D*^ mice and lysed using collagenase P (Roche, Basel, Switzerland) and DNase I (Worthington Biochemical Corp., Lakewood, NJ, USA). The lysed pancreatic ductal cells were seeded in matrigel matrix (Corning, Corning, NY, USA) and grown in culture medium containing Advanced DMEM/F12, B27^™^ Supplement (Gibco), GlutaMAX^™^ (Gibco), R-spondin1, mEGF (Peprotech, Cranbury, NY, USA), mNoggin (Peprotech), hFGF10 (Peprotech), N-acetylcysteine (Sigma-Aldrich), A83-01 (Tocris, Bristol, UK), and nicotinamide (Sigma-Aldrich). Lentiviral CRISPR/Cas9 was used to generate *Galc knockout (*KO) mouse pancreatic organoids. The sgRNA targeting *Galc* was cloned into the lentiCRISPRv2 vector. LentiCRISPRv2-sgGalc, pMD2G, and psPAX2 were transfected into 293FT. The lentiviruses were collected from the cells and concentrated using a Lenti-X^™^ concentrator (Takara, Shiga, Japan). Lentiviruses were transduced into dissociated single cells using TrypLE (Gibco). The transduced cells were recovered in a matrigel matrix and selected using 2 µg/μL puromycin. The generation of *Galc* KO mouse pancreatic organoids was confirmed using semi-quantitative reverse transcription polymerase chain reaction (RT-PCR) with the following primers: Galc_F:5ʹ-AGG TCT CCA GCG AGT GAG AAT CAT AG-3,ʹ Galc_R:5ʹ-TGT GTG AGC TGA TAC CCA GAT AGG AG-3.ʹ To analyze ubiquitinated proteins in mouse pancreatic organoids, the cells were treated with 10 μM MG132 (Enzo, USA) for 24 h. For immunoblotting, the concentration of the extracted organoid protein was measured using the bicinchoninic acid (BCA)^™^ assay (Pierce, Celbio, Milan, Italy) and bovine serum albumin was used as the standard. Equal amounts of the protein extracts were electrophoresed on 10% sodium dodecyl sulfate–polyacrylamide gels and electro-blotted onto polyvinylidene difluoride membranes (Millipore SPA, Milan, Italy). The membranes were then incubated for 1 h at room temperature with blocking solution consisting of 5% skim milk in Tris-buffered saline solution and Tween 20 [TBST; 100 mmol/L Tris (pH 7.5), 0.9% NaCl, and 0.1% Tween 20] and probed overnight at 4 °C using anti-mTOR (Cell Signaling), anti-phospho-mTOR(Cell signaling), anti-rictor (Cell Signaling), anti-LC3B (Cell Signaling), anti-ubiquitin (Cell Signaling), anti-SQTM1/p62 (Abcam), and anti-beta actin (Abcam) antibodies (1:1000 in blocking solution). Horseradish peroxidase-conjugated IgG (1:5000 in blocking solution; Cell Signaling) was used to detect specific proteins. Immunodetection was performed using chemiluminescent substrates (Pierce).

### Immunostaining and in situ hybridization

For the immunofluorescence assay, organoids were isolated from the matrigel using cell recovery solution (Corning) and washed with cold PBS. The isolated organoids were fixed in 4% paraformaldehyde for 1 h, permeabilized in 1% PBS-T (Triton X-100) for 1 h and blocked for 1 h at room temperature in a blocking solution (3% bovine serum albumin in 0.2% PBS-T). The samples were incubated overnight with primary and secondary antibodies (Additional file 5: Table S2) at 4 °C. Then, they were mounted using the VECTASHIELD antifade mounting solution with 4′,6-diamidino-2-phenylindole (DAPI; Vector Laboratories, Burlingame, CA, USA) and imaged using a Zeiss LSM 700 confocal microscope (Zeiss, Oberkochen, Germany). The images were processed using ImageJ software.

For immunohistochemistry (IHC) and in situ hybridization, the fixed organoids were embedded in paraffin blocks. IHC staining was performed using the OptiView DAB IHC detection kit (Ventana, Oro Valley, AZ, USA), according to the manufacturer's instructions. The Ki67 antibody (Invitrogen, Waltham, MA, USA) was used for IHC, and a mouse *Galc* probe (NM_008079.4) was used for in situ hybridization. All the cells were analyzed using the QuPath image analyzer.

### Measuring autophagosome and autolysosome

Organoids were isolated from the matrigel and dissociated into single cells. FUW-mCherry-GFP-LC3 plasmids (Addgene plasmid # 110060) were transduced in the cells using lentiviruses. The transduced cells were then embedded and recovered in matrigel for 48 h. The organoids were fixed in 4% paraformaldehyde for 1 h and mounted using VECTASHIELD Antifade mounting solution with DAPI. Imaging was performed using the DeltaVision system (Applied Precision/GE Healthcare, Issa-quah, WA, USA) and processed using the ImageJ software.

### Patient-derived organoid culture

Human PDAC specimens were homogenized using a GentleMACS^™^ tissue dissociator and a human tumor dissociation kit (Miltenyi Biotec, Bergisch Gladbach, Germany) according to the manufacturer's instructions. After filtering using a 70 μm strainer, the suspended cells were plated on matrigel (Corning) and grown in complete medium: advanced DMEM/F12 supplemented with GlutaMAX^™^, containing 10 mM HEPES, antibiotic antimycotics, B27 supplement, N2 supplement (all from Thermo Fisher Scientific, Waltham, MA, USA), 1 mM n-acetylcysteine, 10 mM nicotinamide (all from Sigma-Aldrich), 60 ng/mL murine Wnt-3a, 500 ng/mL human R-spondin 1, 10 nM human gastrin, 50 ng/mL human Noggin, 50 ng/mL human epidermal growth factor (EGF), 100 ng/mL, human fibroblast growth factor 10 (FGF10), 0.5 μM A83-01 (all from Peprotech), 1 × Primocin (InvivoGen, San Diego, CA, USA), and 10 μM Y-27632. The organoids were cultured in a 37 °C humidified incubator, and the culture medium was partially changed twice a week. Resuspended PDAC cells were seeded into 384-well plates (500 cells/well) with technical duplicates and treated with therapeutic drugs including gemcitabine, nab-paclitaxel, irinotecan and Olaparib (all from Selleckchem) for seven days. Cell viability was accessed using an adenosine triphosphate monitoring system based on firefly luciferase (ATPlite 1step; PerkinElmer) and estimated using the EnVision multilabel reader (PerkinElmer). The relative cell viability for each dose was obtained after normalization with dimethyl sulfoxide (DMSO) per plate.

### Statistical analysis

A linear regression model was used to assess the association between the PPV of each gene and phenotypic characteristics, with a significance cut-off of P < 0.05.

$$\mathrm{glm} (\mathrm{n} \sim \mathrm{germline\, variants} + \mathrm{gender} + \mathrm{age},\mathrm{ family} ="\mathrm{binomial}")$$

where n = case (1) or control (0), germline variants = number of samples that carry rare pathogenic germline variants, gender = male (0) and female (1), and patient age at diagnosis were used as inputs for the regression model. Chi-squared and Fisher's exact tests were used to evaluate the associations between categorical variables. They were applied for the most prevalent comparisons for testing the association of variant incidence according to the disease phenotype. Independent t-test and one-way analysis of variance were used to assess the association between continuous variables. Survival analysis was performed using the Kaplan–Meier method. All statistical analyses were performed using either SPSS (version 25.0; IBM SPSS Statistics, Armonk, NY: IBM Corp) or R (http://www.r-project.org/).

## Results

### Baseline characteristics of the patients

The baseline characteristics of the 418 patients with PDAC are presented in Table 1. Patients’ ages ranged from 35 to 87 years, with a median age of 65 years. Among these, 243 (58.1%) were male, and 175 (41.9%) were female. The median value of body mass index was 22.7 kg/m^2^, and 146 patients (34.9%) had diabetes mellitus at the time of diagnosis. The median values of carcinoembryonic antigen and carbohydrate antigen 19-9 were 2.4 ng/mL and 150.6 U/mL, respectively. According to the 8th edition of the AJCC on Cancer staging system, 78 (18.7%), 123 (29.4%), 112 (26.8%), and 105 (25.1%) patients had stage I, II, III, and IV disease, respectively. Univariate analysis revealed a significant association between the stage and overall survival (OS), with median OS for stages I, II, III, and IV being 37.0, 24.0, 18.7, and 10.6 months (P < 0.001), respectively, implying that our cohort was a general pancreatic cancer cohort rather than a biased cohort with an unusual disease course. In addition, we recruited 845 individuals without any history of cancer through a health examination center of SNUH over 3 years who were grouped as the control set (named CFNC), respectively. These samples were selected only from those aged 50 years or older (age ranged from 51 to 91 years).

**Table 1:** Baseline characteristics of patients with pancreatic ductal adenocarcinoma

Variables	
Age, years, median (range)	65 (35–87)
Sex, no. (%)	
Male	243 (58.1)
Female	175 (41.9)
BMI, kg/m^2^, median (range)	22.7 (14.3–32.3)
DM, no. (%)	
No	272 (65.1)
Yes	146 (34.9)
Smoking, no. (%)	
No	296 (70.8)
Yes	122 (29.2)
Alcohol, no. (%)	
No	278 (66.5)
Yes	140 (33.5)
CEA, ng/mL, median (range)	2.4 (0–880)
CA 19-9, U/mL, median (range)	150.6 (0–140,000)
AJCC 8th stage of cancer, no. (%)	
I	78 (18.7)
II	123 (29.4)
III	112 (26.8)
IV	105 (25.1)
CPG carrier, no. (%)^a,c^	15 (3.6)
LSD gene carrier, no. (%)^b,c^	34 (8.1)
Non-carrier, no. (%)	371 (88.7)

*SD* standard deviation, *BMI* body mass index, *DM* diabetes mellitus, *CEA* carcinoembryonic antigen, *CA 19-9* carbohydrate antigen 19-9, *AJCC* The American Joint Committee on Cancer, *CPG* cancer predisposition genes, *LSD* lysosomal storage disease

^a^Gene; CHEK2, BRCA2, COL7A1, BRCA1, ATM, KRAS, TP53

^b^Gene; GALC, HEXB, NPC1, IDUA, PSAP, MAN2B1, GAA, ARSA, HEXA, SGSH, NAGLU, MCOLN1, HYAL1, GUSB, GNPTG

^c^Two patients were carriers of both the CPG and LSD genes

### Enrichment of LSD germline variants in the PDAC cohort

Thirty-seven PPVs, including SNVs and indels, were detected in 21 of the 42 LSD genes (*ARSA, GAA, GALC, GLB1, GNPTAB, GNPTG, GUSB, HEXA, HEXB, HGSNAT, HYAL1, IDUA, MAN2B1, MCOLN1, NAGLU, NPC1, PSAP, SGSH, SMPD1, SUMF1,* and *TPP1*; Additional file 4: Table S1)*.* Most variants were PTVs (24 of 37) and 48.7% were clinically proven pathogenic variants of ClinVar [30] (pathogenic or likely pathogenic; 18 of 37). Specific information on individual variants found in PDAC are listed in Additional file 6: Table S3. Among those, two typical variants in *GALC* were identified most frequently, although not statistically significant; rs138577661 was found in 2.4% PDAC patients (10 out of 418 PDAC patients), which was twice as many as that in the CFNC group (1.2%, 10 out of 845 individuals; *P* = 1.05 × 10^–1^ in Chi-squared test), and rs200607029 was found in 1.0% PDAC patients (4 out of 418 PDAC patients) compared to 0.4% in the CFNC group (3 out 845 CFNC group; *P* = 1.75 × 10^–1^ in Chi-squared test).

Overall, 34 Korean patients with PDAC (34/418, 8.13%) harbored at least one genomic variant, whereas 36 germline carriers (36/845, 4.26%) were identified among CFNC (P = 6.92 × 10^–3^ by Chi-squared test). The most frequently mutated gene in *GALC* (3.6%) was the most frequently mutated gene in Korean patients with PDAC, followed by *HEXB* (1.2%), *GAA* (0.5%), and *NAGLU* (0.5%; Additional file 4: Table S1). We also estimated the effect of PPV using a regression model after adjusting for gender and age using samples from WES data (418 patients with PDAC and 352 controls to avoid variant selection bias due to technical differences). Results revealed PDAC enrichment of LSD variants with Log_2_ odds ratio (OR) of 1.65 (P = 3.08 × 10^–3^). Of the 418 Korean patients with PDAC, 15 (3.6%) had CPG variants, including *BRCA1/2*, *ATM*, and *COL7A1*, and two patients harbored rare pathogenic germline variants in both LSD genes and CPGs (Table 1).

### Early onset of PDAC in patients with LSD gene carriers

We tested whether pathogenic germline variant carriers in LSD gene developed PDAC at a younger age than non-carriers, similar to that observed in the well-known CPG carriers who are more likely to develop cancer at a younger age (Additional file 1: Figure S1 and Table 2). Two patients with both CPG and LSD gene variants were classified as CPG carriers, and the characteristics of each study group were compared. The mean ages at diagnosis of PDAC in CPG carriers and LSD carriers were 60.4 and 61.7 years, respectively, which were significantly lower than that in patients without CPG or LSD variants (65.3 years; P = 0.025). In addition, for all LSD gene carriers (n = 34), including two who also have CPG mutations, the mean age at diagnosis of PDAC was 61.5 years, which was significantly lower than 65.3 years for non-carriers of either LSD or CPG (n = 371) (P = 0.031). This indicates that LSD carriers could be one of the high-risk populations who showed early development of PDAC. Other than age, clinical characteristics, including tumor stage, did not differ significantly according to CPG or LSD gene carrier status (Table 2).

**Table 2:** Patient characteristics according to CPG or LSD genes carrier status

Variable	CPG carrier^a^	LSD gene carrier^a^	Non-carrier	P-value
	n = 15	n = 32	n = 371	
Age, years, mean ± SD	60.3 ± 7.2	61.7 ± 11.5	65.3 ± 9.54	0.025
Sex				
Male	9 (2.2)	22 (5.3)	212 (50.7)	0.438
Female	6 (1.4)	10 (2.4)	159 (38.0)	
BMI, kg/m^2^, mean ± SD	23.0 ± 2.8	22.3 ± 2.6	22.8 ± 3.0	0.628
DM, no. (%)				
Yes	5 (1.2)	14 (3.3)	127 (30.4)	0.551
No	10 (2.4)	18 (4.3)	244 (58.4)	
Smoking, no. (%)				0.597
Yes	3 (0.7)	11 (2.6)	108 (25.8)	
No	12 (2.9)	21 (5.0)	263 (62.9)	
Alcohol, no. (%)				0.809
Yes	4 (1.0)	10 (2.4)	126 (30.1)	
No	11 (2.6)	22 (5.3)	245 (58.6)	
CEA, ng/mL, mean ± SD	9.7 ± 25.5	4.0 ± 6.5	9.9 ± 55.2	0.872
CA 19–9, U/mL, mean ± SD	555.0 ± 1136.3	4092.3 ± 8559.6	2677.3 ± 11,499.8	0.588
AJCC 8th stage, no. (%)				0.840
I	4 (1.0)	8 (1.9)	66 (15.8)	
II	4 (1.0)	11 (2.6)	108 (25.8)	
III	4 (1.0)	7 (1.7)	101 (24.2)	
IV	3 (0.7)	6 (1.4)	96 (23.0)	

*CPG* cancer predisposition genes, *LSD* lysosomal storage disease, *SD* standard deviation, *BMI* body mass index, *DM* diabetes mellitus, *CEA* carcinoembryonic antigen, *CA 19-9* carbohydrate antigen 19-9, *AJCC* The American Joint Committee on Cancer

^a^Two patients who were carriers of both the CPG and LSD genes were classified as CPG carriers

### Defective lysosomal function in *Kras*^*G12D*^*/Galc* knockout mouse-derived pancreatic organoids

To investigate the effects of autophagy regulation and lysosomal function on PDAC development, a lysosomal enzyme GALC was eliminated from *Kras*^*G12D*^ mice-derived pancreatic organoids using CRISPR/CRISPR-associated protein 9 (Cas9). As mentioned, *Galc* variants were the most frequent LSD PPV in our cohort. *Galc* expression was reduced in *Kras*^*G12D*^*/Galc* knockout (KO) organoid, which was confirmed using semi-quantitative RT-PCR (Additional file 2: Fig. S2a), RNA sequencing (as the median expression value of *Kras*^*G12D*^ and *Kras*^*G12D*^*/Galc* KO organoid: 5.13 vs. 1.04, *P* = 0.05 in Wilcoxon rank sum test (Additional file 2: Fig. S2b), and in situ hybridization (Additional file 2: Fig. S2c, d).

**Fig. 1:**
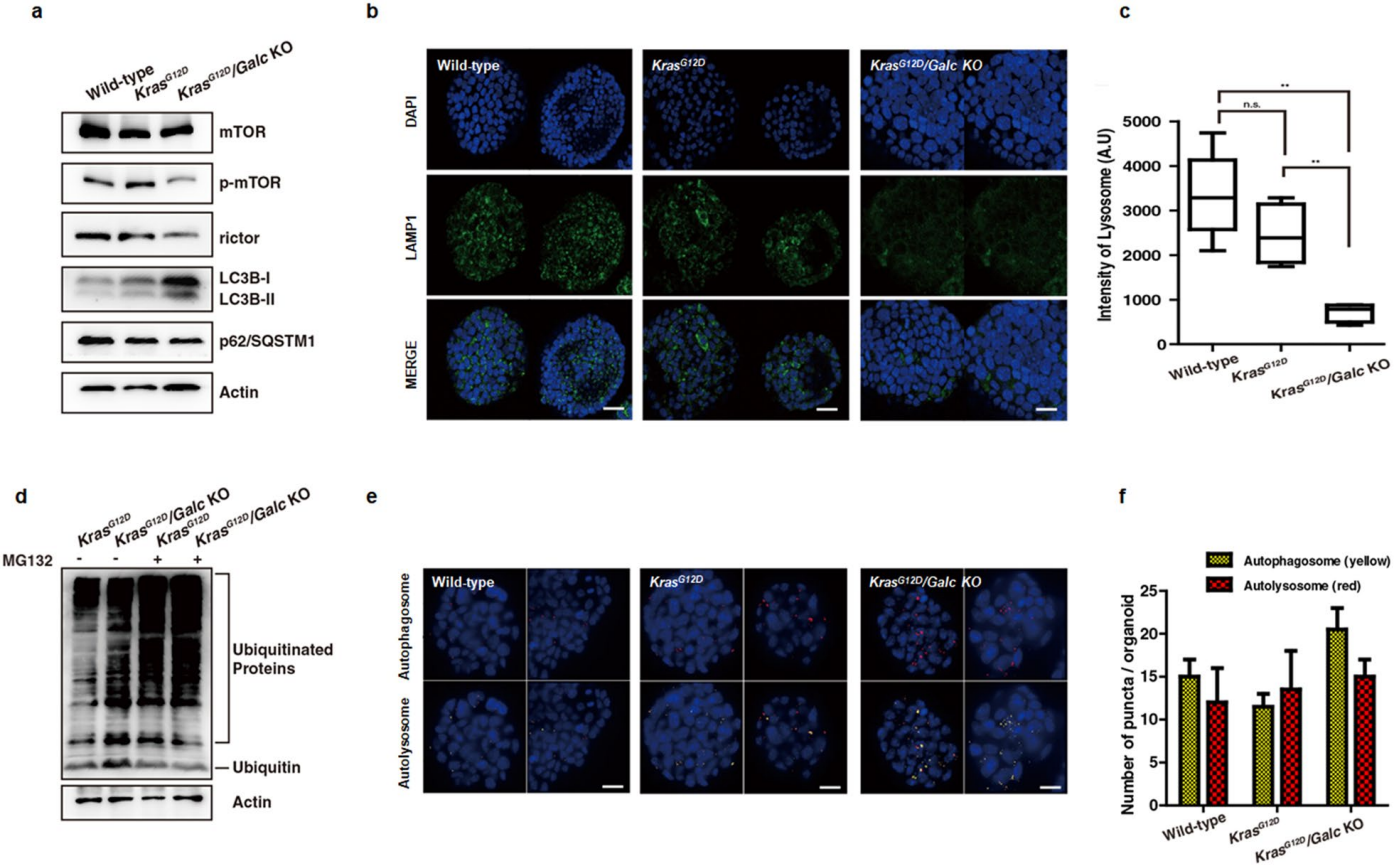
Increased autophagic flux and impaired lysosomal function in *Kras*^*G12D*^*/Galc* knockout mouse pancreatic organoids. Pancreatic organoids were cultured from pancreatic duct of wild-type and *Kras*^*G12D*^ mice, and *Kras*^*G12D*^*/Galc* knockout (KO) pancreatic organoids were generated by eliminating *Galc*. **a** Expression of mTOR, phosphorylated mTOR (p-mTOR), rictor, LC3BI/II, and p62 was examined in each pancreatic organoid using immunoblotting. **b** and **c** The intensity LAMP1, a lysosomal marker, was measured after immunofluorescent staining. P** < 0.01. **d** Proteasomal degradation levels were detected before and after treatment with MG132, a proteasome inhibitor, for 24 h. **e** and **f** Fluorescence-labeled autophagosomes and autolysosomes were detected and counted in mouse pancreatic organoids. P** < 0.01, P*** < 0.001

The autophagic flux in *Kras*^*G12D*^ organoids after *Galc* depletion was observed via western blotting of LC3B-II, a standard autophagosome marker (LC3B-I is lipidated upon autophagy activation to form LC3B-II), and SQSTM1/p62, a receptor involved in autophagy [38, 39]. LC3B-II level increased slightly in *Kras*^*G12D*^ organoids and markedly in *Kras*^*G12D*^*/Galc* KO organoids (Fig. 1a). In contrast, SQSTM1/p62 level did not differ significantly between *Kras*^*G12D*^ organoids and *Kras*^*G12D*^*/Galc* KO organoids. Then, we assessed the levels of mammalian target of rapamycin (mTOR), as mTORC2 (mTOR-rictor component) negatively regulates autophagy [40]. The results showed that the level of phospho-mTOR (p-mTOR) decreased considerably in the *Kras*^*G12D*^*/Galc* KO organoids, while that of mTOR was intact. Moreover, rictor (rapamycin-insensitive companion of mTOR), which is a component of mTORC2, was downregulated in *Kras*^*G12D*^*/Galc* KO organoids, suggesting that inactivation of mTORC2 led the autophagic flux (Fig. 1a).

In addition, lysosomal function was examined via immunostaining of lysosome-associated membrane protein 1 (LAMP1). The results showed that the intensity of LAMP1 decreased considerably in *Kras*^*G12D*^*/Galc* KO organoids (Fig. 1b), as represented in the bar graph (Fig. 1c), indicating that *Galc* depletion in *Kras*^*G12D*^ causes lysosomal dysfunction.

As autophagic activity increased with defective lysosome function, we investigated the formation of autophagosomes and autolysosomes in wild-type, *Kras*^*G12D*^, and *Kras*^*G12D*^*/Galc* KO organoids. We transfected the mCherry-GFP-LC3 plasmid in three different types of organoids to visualize the formation of authophagosomes and autolysosomes, and their numbers were analyzed. Owing to the acidity of GFP, autopahgosomes are marked by both mCherry and GFP (yellow) signals, while the autolysosomes were marked with only mCherry (red) [41]. The number of autophagosomes was higher in *Kras*^*G12D*^*/Galc* KO organoids than in the wild-type or *Kras*^*G12D*^ organoids, whereas the number of autolysosomes did not differ among organoids of the three different genotypes (Fig. 1e, f). Furthermore, we found that the accumulation of ubiquitinated proteins was enhanced in *Kras*^*G12D*^*/Galc* KO organoids compared to that in the *Kras*^*G12D*^ organoids both before and after treatment with MG132, a proteasome inhibitor, for 24 h (Fig. 1d).

These results suggested that defective GALC in *Kras*^*G12D*^ organoids enhanced autophagic flux and promoted autophagosome formation. However, autolysosome formation and degradation did not follow because of lower lysosomes and lysosomal dysfunction, causing accumulation of ubiquitinated proteins in *Kras*^*G12D*^*/Galc* KO organoids.

### Increased proliferation of *Kras*^*G12D*^*/Galc* knockout mouse-derived pancreatic organoids

**Fig. 2:**
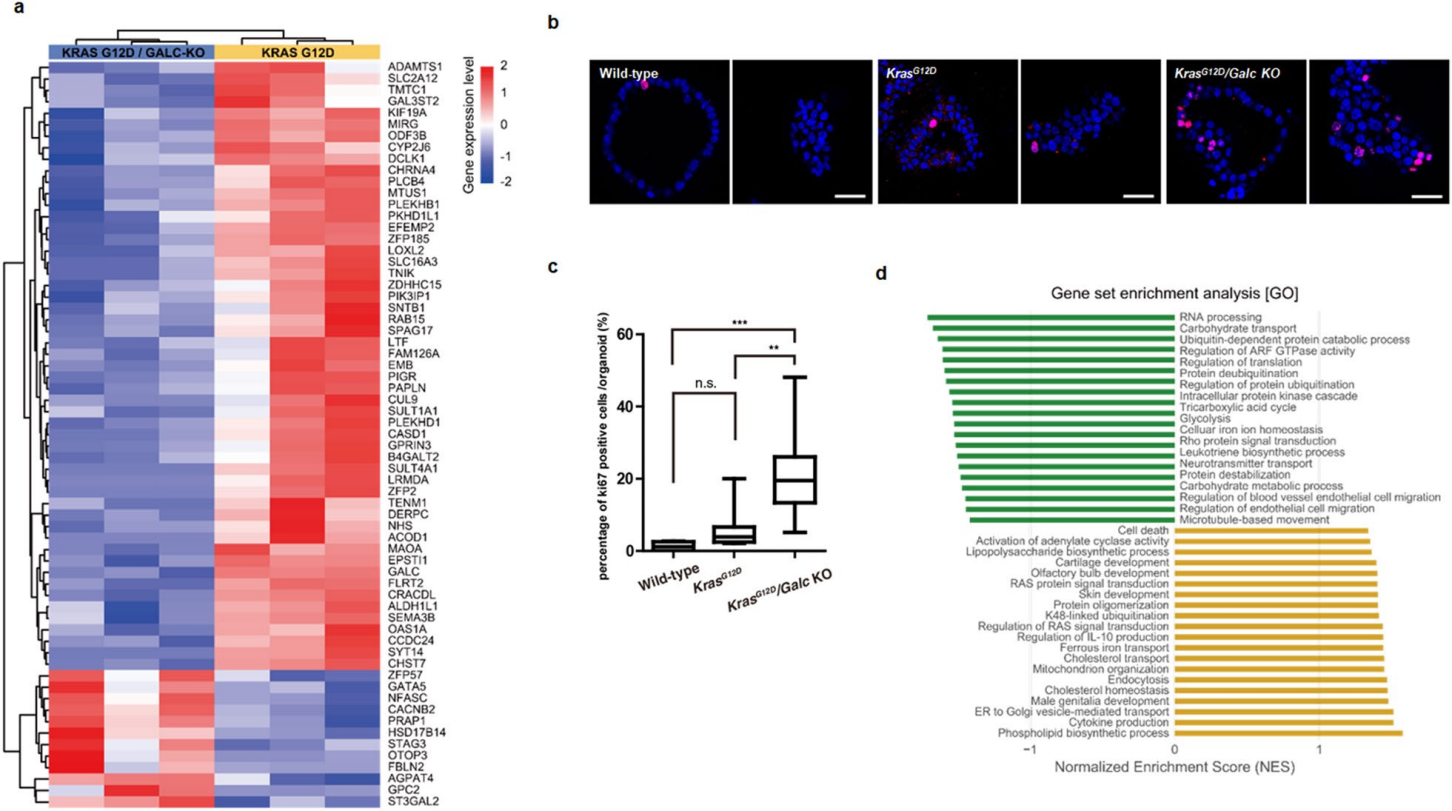
Increased proliferation and profile of differentially expressed genes in *Kras*^*G12D*^*/Galc* knockout mouse-derived pancreatic organoids. **a** Heatmap of RNA sequencing showing differentially expressed genes between *Kras*^*G12D*^ knockout (KO) organoids and *Kras*^*G12D*^*/Galc* KO organoids. **b** and **c** Proliferation of organoids was visualized using Ki-67, a proliferation marker, using immunofluorescence assay under confocal fluorescence microscope, and the number of Ki-67 was represented in a bar graph. P** < 0.01, P*** < 0.001. **d** The biological process, cellular components and molecular function were identified from GO enrichment analysis. GO, Gene Ontology

To understand the regulatory mechanism underlying lysosomal dysfunction that affect pancreatic tumorigenesis, we analyzed the gene expression patterns of *Kras*^*G12D*^ and *Kras*^*G12D*^*/Galc* KO pancreatic organoids using RNA-seq. In DEG analysis, the majority (81.5%) of the genes, including *MIRG, PIGR, SYT14, PLEKHD1*, and *SLC2A1*, were found to be downregulated in *Kras*^*G12D*^*/Galc* KO organoids. In addition, low expression of monoamine oxidase A (*MAOA*), a key factor that regulates apoptosis/autophagy by targeting the repressor element-1 silencing transcription factor (*REST1*), was identified in *Kras*^*G12D*^*/Galc* KO organoids. In contrast, the expression of genes, including *STAG3, GATA5,* and *CACNB2,* which are associated with cell proliferation or tumorigenesis, and *PRAP1*, which is primarily involved in the inhibition of tumor cell apoptosis, increased in mouse *Kras*^*G12D*^*/Galc* KO pancreatic organoids (Fig. 2a).

To assess cell proliferation, we examined Ki-67 expression in mouse pancreatic organoids. The number of Ki-67-positive proliferating cells was considerably higher in *Kras*^*G12D*^*/Galc* KO organoids and lower in wild-type and *Kras*^*G12D*^ organoids (Fig. 2b, c, Additional file 3: Fig. S3). Furthermore, GSEA revealed that *Kras*^*G12D*^*/Galc* KO organoids were strongly associated with *RAS* signaling, cytokine production, and cell death in GO terms by MSigDB. However, regulation of translation, RNA processing, protein ubiquitination, and glycolysis were significantly downregulated in the *Kras*^*G12D*^*/Galc* KO pancreatic organoids (Fig. 2d). In summary, transcriptomic analysis of *Galc* KO mouse-derived pancreatic organoid indicated that GALC dysfunction resulted in impaired autophagic function and markedly enhanced cell proliferation.

### Enhanced autophagic flux in PDAC cell lines with LSD gene variants

**Fig. 3:**
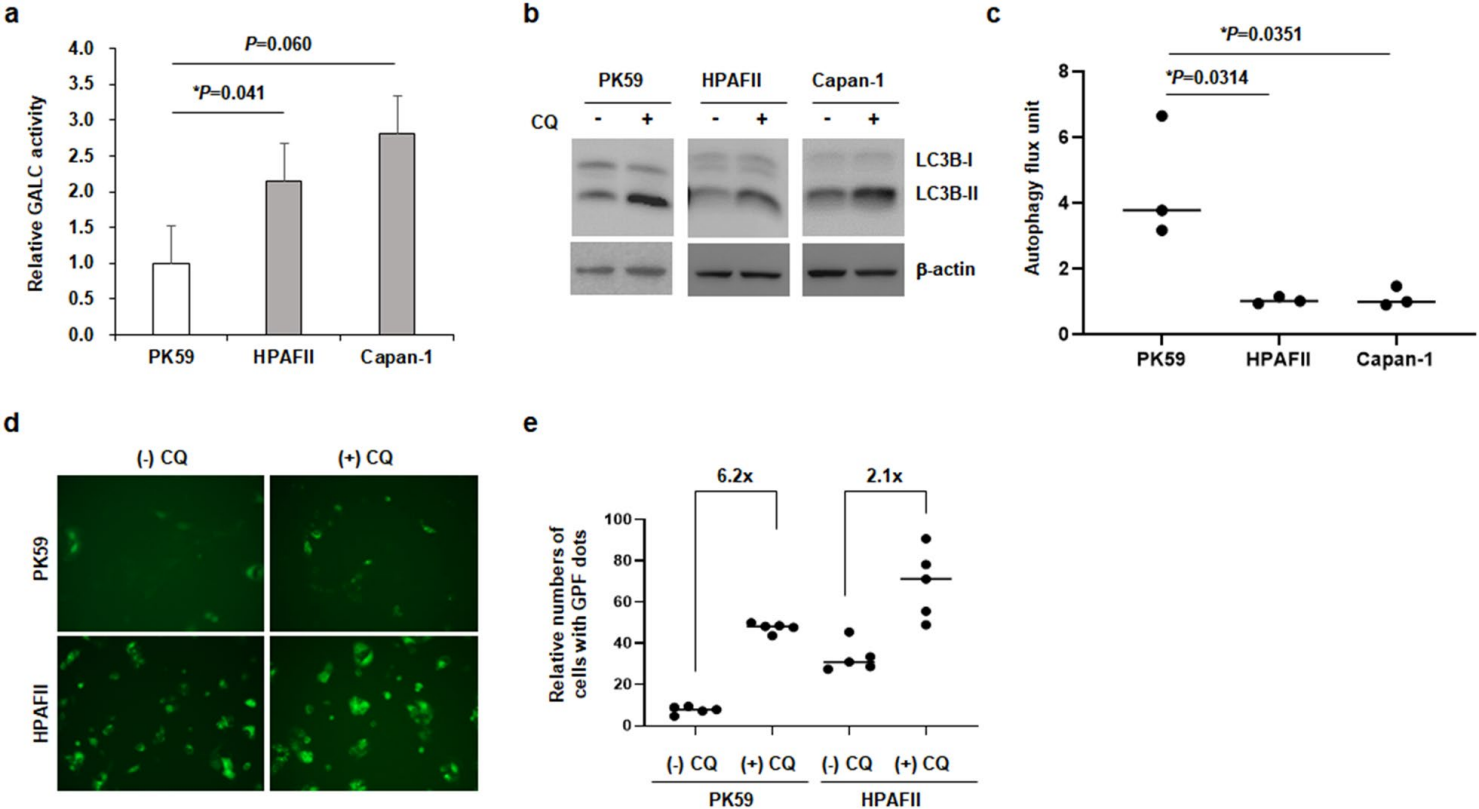
Enhanced autophagic flux in PDAC cells with *Galc* variant. **a** Endogenous activity of GALC in PK59 (*Galc* variant), HPAFII and Capan-1 (*Galc* wild-type) was measured using lysosomal GALC analysis. **b** PK59, HPAF-II and Capan-1 cells were treated with or without 100 μM chloroquine (CQ) for 1 h to monitor autophagy flux. Expression levels of LC3B-I/II in PDAC cell lines was detected in three independent immunoblots. **c** Autophagy flux unit represents a buildup of LC3B-II with CQ treatment. Densitometric analysis was performed to measure the band intensity of LC3B using ImageJ. **d** PK59 and HPAF-II cells that stably expressed GFP-LC3 were treated with CQ for 5 h, and GFP-LC3 dots were measured using a fluorescence microscopy. **e** Number of GFP-LC3 dots were counted manually from the fluorescent images. Indicated numbers above the graphs show an increase in the ratio of LC3 dots under CQ treatment condition relative to that under no treatment condition

Next, we examined the mechanisms underlying autophagy regulation in PDAC cells with LSD PPVs. The endogenous activity of lysosomal GALC enzyme was lower in PK59 (*Galc* variant) cells than in HPAFII and Capan-1 (*Galc* wild-type) cells (Fig. 3a). Furthermore, LC3B-II was observed after treatment with CQ, an inhibitor of autophagy, to monitor autophagy flux. The increase in the amount of LC3B-II after CQ treatment was prominent in PK59 cells (Fig. 3b and c). Moreover, we generated PK59 and HPAFII cells stably expressing GFPto a-LC3 and monitored the number of LC3-positive cells after CQ treatment. The relative increase in GFP-expressing cells was more pronounced in PK59 cells than that in HPAFII cells (Figs. 3d and e). These results indicated enhancement of autophagic flux in PDAC cells with low GALC activity, which is associated with *Galc* variants.

### Drug responses in PDAC cells with LSD germline variants

**Fig. 4:**
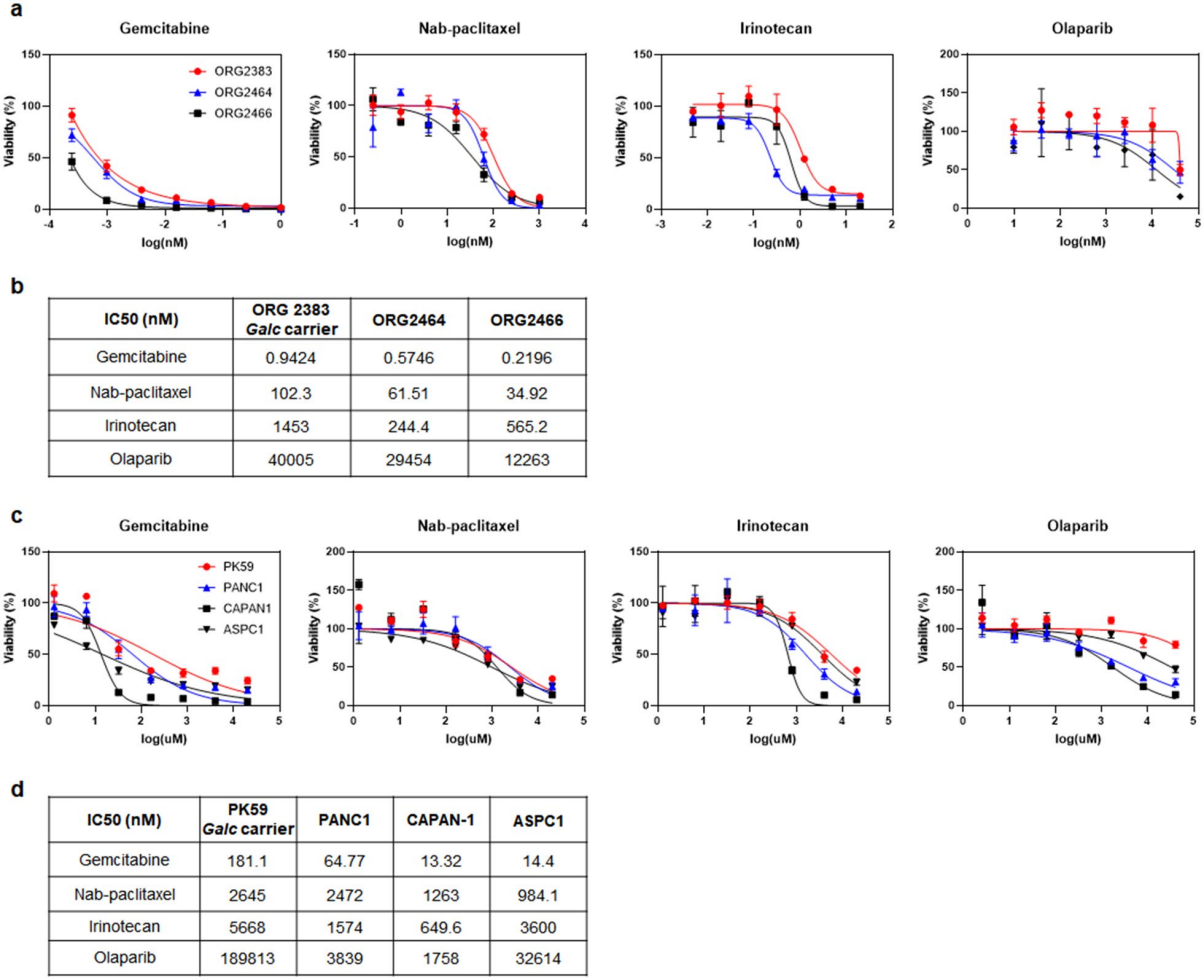
Response to therapeutic drugs in human PDAC patient-derived organoids and PDAC cell lines. **a** and **b** Human PDAC PDOs and **c** and **d** PDAC cell lines were treated with gemcitabine, nab-paclitaxel, irinotecan, and Olaparib for 7 days. Cell viability was assessed using adenosine triphosphate monitoring (in triplicate). Dose–response curves were fitted, and the half-maximal inhibitory concentration (IC_50_) values (nM) were calculated. ORG2383 and PK59 are *Galc* carriers

We performed a drug test using PDAC PDOs and PDAC cell lines to examine their responses to treatment according to LSD PPV carriers (Fig. 4). PDAC PDOs and PDAC cell lines with or without *GALC* variants were treated with representative therapeutic drugs for PDAC, including gemcitabine, nab-paclitaxel, irinotecan, and Olaparib for 7 days, and the viability was assessed for calculating the half maximal inhibitory concentration (IC50). We observed that *Galc* carriers ORG2383 and PK59 showed higher IC50 values than other PDAC PDOs (Fig. 4a and b) and PDAC cell lines (Fig. 4c and d). Thus, *Galc* carriers tended to be more resistant to drugs used for treating PDAC.

### Evaluation of pathways associated with LSD germline variants in human PDAC PDOs

**Fig. 5:**
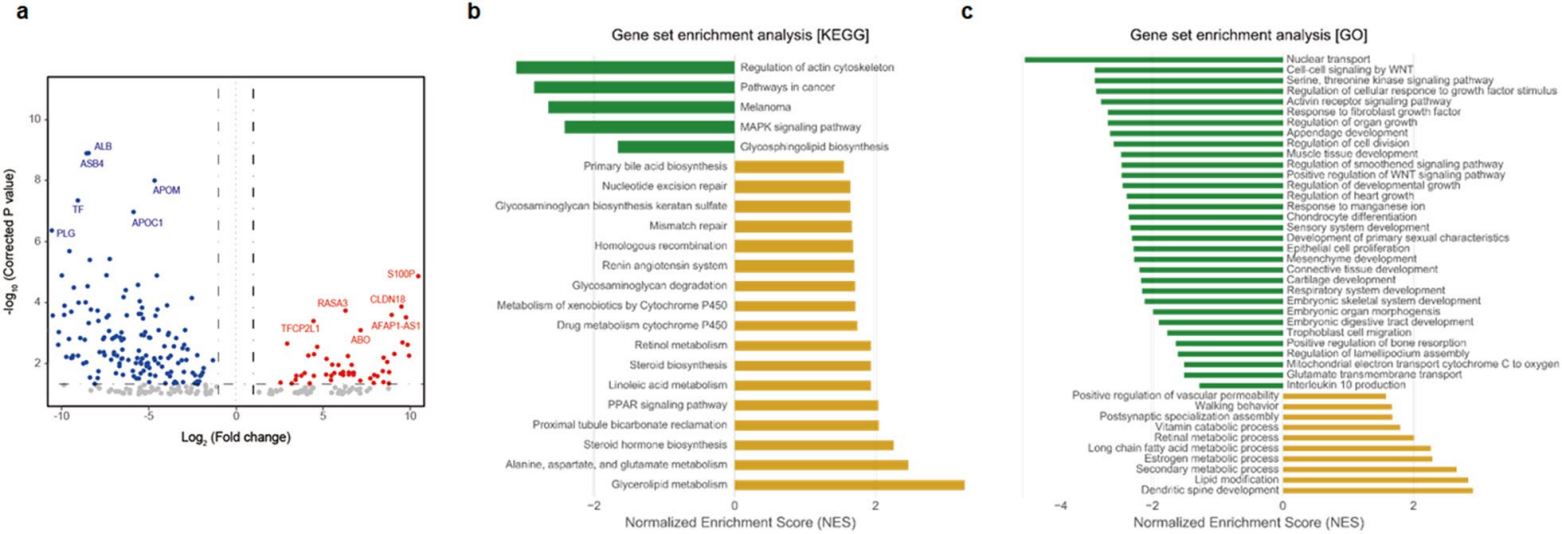
Evaluation of expression profile in PDOs derived from *Galc* carriers with PDAC. **a** Volcano plot depicting the differentially expressed genes between *Galc* carrier PDAC PDOs (n = 2) and non-carrier PDAC PDOs (n = 27). Dashed vertical lines represent the threshold of log2 fold-change (range of ≥ 1 and ≤  − 1) and the horizontal line represents the threshold of statistical significance (adjusted *P* ≤ 0.05). The colors indicate whether the differentially expressed genes were significantly down-regulated (log2 fold-change ≤  − 1; blue), or up-regulated (log2 fold-change ≥ 1; red). **b** and **c** Biological process, cellular components and molecular function were identified from the GO and KEGG enrichment analyses. *GO* gene ontology, *KEGG* Kyoto encyclopedia of genes and genomes

To investigate the functional consequences of lysosomal dysfunction in established PDAC, we conducted RNA-seq of LSD PPV carriers (n = 2, both *GALC* heterozygote carriers) and non-carriers (n = 27) using our PDAC PDOs. Differential expression analysis identified 240 DEGs, of which 68 were upregulated, including *S100P, AFAP1-AS1, TFCP2L1*, and *RASA3,* and 172 were downregulated, including *APOM, APOC1, ASB4, TF*, and *PLG* in LSD carriers [Log_2_FoldChange > 1, false discovery rate (FDR) < 0.05] (Fig. 5a) compared to that in LSD non-carriers. However, the small sample size might be a limitation of this study, as it might affect the reproducibility of the results of DEG analysis. Interestingly, GSEA analysis to test the carcinogenic processes depending on *LSD* gene variation showed that amino acid biosynthesis-related pathways, including primary bile acid biosynthesis, steroid hormone biosynthesis, linoleic acid metabolism, alanine, aspartate, and glutamate metabolism, and glycerolipid metabolism were strongly upregulated in LSD carriers in KEGG (Fig. 5b). Furthermore, we identified upregulation of the DNA repair system (nucleotide excision repair, mismatch repair, and homologous recombination) in LSD carriers. In addition, metabolic upregulation was confirmed using the GO analysis from a biological perspective that occurs within cells in LSD carriers (Fig. 5c). However, most cell-to-cell signaling and organ development pathways were downregulated in PDAC PDOs from LSD carriers (Fig. 5c).

## Discussion

Germline mutations in genes are associated with risk of cancer development and may cause early onset cancer [42]. Most genes involved in carcinogenesis have functions related to cell division and proliferation. The present study investigated the oncogenic effects of LSD heterozygosity in patients with PDAC. We evaluated the clinical implications of the rare variants of LSD genes on a large scale PDAC patients and healthy controls, and functional analyses were performed using mouse pancreatic organoids, PDAC cell lines, and PDAC PDOs. First, we found that LSD PPV carriers were enriched in the PDAC population compared to that in the healthy controls and they developed PDAC at a younger age than the non-carriers. Second, we showed paradoxically increased autophagic flux due to impaired autophagolysosome activity, which may contribute to PDAC development, using mouse pancreatic organoids. Third, PDAC PDOs with LSD PPV showed upregulation of metabolic pathways, which supports the idea that genes involved in lysosome function may also contribute to PDAC development via alterations in autophagic activity.

The enrichment of germline LSD PPV was approximately three times (Log_2_OR 1.6–1.8) in the Korean PDAC population compared to that in the healthy controls. This enrichment is also accompanied by a younger age of onset of PDAC by 3.6 years compared to that of non-LSD PPV carriers, similar to that observed with other CPGs. A previous study has reported the enrichment of six representative germline PPVs, including *BRCA1/2* and *TP53*, in the PDAC population compared to that in the general population, with ORs ranging from 2.58 to 12.3 (all ORs were more significant than five except for 2.58 for BRCA1) [6] implying that the oncogenic potential of germline LSD PPV was weaker than that of conventional CPGs. Dysfunction of lysosomes and autophagy, which unlocks phenotypic plasticity, is an emerging hallmark of cancer [43]. Thus, LSD PPV may be used as a biomarker for distinguishing the high-risk population for PDAC after validation.

Technically, due to the genetic diversity of rare disease variants [44, 45], ethnic factors should be addressed in this type of analysis. In fact, several studies have revealed distinct germline predisposition according to ethnicity in many diseases [46–49]. We have eliminated this issue by focusing on a single ethnic Korean population for the germline PPV analysis. As Koreans have relatively homogenous genetic background, comparison of PPV frequencies across cohorts is feasible [50]. In the PPV analysis, *GALC, HEXB, GAA,* and *NAGLU* were identified most frequently in the Korean PDAC population. Among these, the contributions of *GALC* PPVs (rs138577661 and rs200607029), which are well-known variants of the Krabbe disease in the East Asian populations, was found to be considerable in our study [51]. The functionality of the rs138577661 variant was confirmed by performing enzymatic testing of a PDAC cell line (PK59) harboring the exact variant. Overall, the variant-level data also suggested the importance of ethnicity in this study on rare PPVs. Hence, extrapolation of our data to other ethnic populations should be performed with caution, and consideration of whether other rare PPVs are implicated in another ethnic population might be required.

Among the various cancers, we selected PDAC for investigation based on the results of a previous study that revealed the possible link between LSD PPV and PDAC [13] and its well-known relation with lysosomes, autophagy [52], and abnormal metabolism [53]. From the perspective of tumor initiation and development, dysfunctional lysosomes impede the autophagolysosomal activity of abnormal cells, resulting in cancer cell survival and growth [54]. From the perspective of cell death, lysosomal or autophagic cell death is not adequate for elimination of cancerous cells [55]. Indeed, evidence suggests that dysfunctional lysosomes contribute to cancer initiation [16, 56]. Our results show conclusively that *Galc* knockdown promotes cell proliferation (elevated Ki-67) in cooperation with *Kras* mutations in mouse pancreatic organoids. In addition, we observed that autophagolysosome function decreased, which subsequently increased autophagic flux as a feedback mechanism, suggesting alteration of autophagic activity with *GALC* downregulation. Considering the cellular function of lysosomes, we hypothesized that cooperation between oncogenic mutations such as *RAS* and lysosomal dysfunction is necessary for carcinogenesis. However, as we did not observe all the features of cancer in the mouse organoids, future studies using mice with knockdown of lysosome-related genes are necessary to confirm spontaneous cancer development. Furthermore, manipulation of specific variants such as rs138577661 is worthwhile to understand the carcinogenic mechanism of specific variants related to lysosome dysfunction.

However, once a tumor is established, autophagy plays a vital role in maintaining the cancer in many ways. In particular, for PDAC, nutrient-poor and hypoxic conditions allow cancer cells to thrive in harsh environments, which render autophagy important for recycling nutrients [53]. In addition, in PDAC, macropinocytosis plays a role similar to that of autophagy. Both autophagy and macropinocytosis rely on lysosomes for the final degradation of products. Macropinocytosis is induced by the blockade of autophagic activity in PDAC via *NRF2* induction [57, 58], which is indicative of their compensatory roles. We interpreted that the increased autophagic flux in PDAC cell lines originates from lysosomal dysfunction, which compensates for the hampered autophagolysosomes. This increased autophagic flux may favor the survival of established cancer cell, with paradoxically increased autophagy and/or macropinocytosis in cancer cells. Furthermore, upregulation of autophagy not only enhances the tumor survival, but also induces drug resistance due to renewal of by cytoplasmic materials, gene repair, alterations in drug concentration and metabolism, and changes in the expression or activities of key proteins. In established cancers, autophagy increases metabolism to inactivate drugs and support drug resistance [59–61]. Moreover, autophagy actively participates in maintaining genomic integrity, as well as in repair processes [62], and the deregulation of DNA repair pathways is associated with the initiation, progression and resistance of cancer cells by promoting genomic instability and mutation [63]. Indeed, RNA-seq analysis revealed that several metabolic pathways and gene repair pathways were upregulated in PDAC PDOs from patients who were LSD PPV carriers, suggesting enhanced metabolic utility and dysregulation of DNA repair system in established PDAC with lysosome dysfunction. Further studies are required to generate direct evidence supporting our interpretations.

Moreover, the LSD PPV carriers tended to be less sensitive to drugs in both PDAC cell lines and human PDAC PDOs. The expression of S100 calcium-binding protein P (S100P) has been associated with drug resistance, metastasis, and poor clinical outcomes [64]. S100P promotes pancreatic cancer growth, survival, and invasion, and its intracellular levels affect resistance to 5-fluorouracil treatment in vitro [65]. In addition, high expression of the long non-coding RNA actin filament-associated protein 1 antisense RNA1 (*AFAP1-AS1*) is associated with poor survival and short-term recurrence in PDAC. Knockdown of *AFAP1-AS1* attenuated PDAC cell proliferation, migration, and invasion [66]. Apolipoprotein M (APOM) can suppress the proliferation and invasion of hepatocellular carcinoma [67], breast cancer [68], and larynx carcinoma [69]. These results are in agreement with upregulation of *S100P* and *AFAP1-AS1* and downregulation of APOM in the RNA-seq data of PDOs carrying LSD PPVs.

From a genetic perspective, our results imply that the heterozygous status of LSD genes is related to cancer development. Accordingly, we checked whether LSD genes follow Knudson’s two-hit hypothesis [70] similar to most CPGs. However, we did not observe any significant loss of heterozygosity in PDAC patients with LSD PPV from CNA analysis using matched tumor sequencing data (data not shown). Our results suggested that lysosomes and autophagy play biphasic roles in the initiation and progression of cancer. Complete loss of autophagy is not suitable for cancer cell survival in the PDAC microenvironment. Notably, we provide evidence showing that pathogenic variants in LSD genes can lead to the development of adult-onset chronic diseases such as PDAC other than the classic LSD phenotype, similar to that observed for the development of Parkinson’s disease and heterozygote carriers of *GBA* rare variants [15].

Finally, many attempts to develop therapeutics targeting autophagy in PDAC are underway, including a clinical trial (NCT03825289) exploring concurrent inhibition of the RAS-RAF-MEK-ERK pathway and autophagy [71–75]. The use of nanodrugs in PDAC have also been actively investigated [76, 77]. As all these modalities are closely related to lysosomal function, our results can act as a cornerstone for developing novel therapeutics for PDAC.

## Conclusion

In conclusion, we showed the fact that genetically defined lysosomal dysfunction is frequently observed in young-onset PDAC. Lysosomal dysfunction may contribute to PDAC development by impairing autophagolysosome activity. In established PDAC, lysosomal dysfunction is closely associated with an increased autophagic flux and upregulated metabolism. This is the first study to report a relationship between lysosome dysfunction and PDAC development. Consistency in our results obtained using multiple methods, including DNA sequencing, RNA-seq analysis, and experiments using knockouts in mouse organoids and patient-derived human organoid, indicate the validity of our findings. We believe that our observations will act as a cornerstone for research on the role of lysosome dysfunction in PDAC. However, as our study has focused on a single ethnic group (Korean), generalization of our results to PDAC in non-Asian populations should be avoided till further investigations are performed. In addition, future studies should aim to understand the complete mechanism underlying lysosomal dysfunction in PDAC carcinogenesis, as we observed increased proliferation of pancreatic cells, and not complete carcinogenesis, after *GALC* knockdown.

### Supplementary Information

**Additional file 1: Figure S1.** Onset age in PDAC patients who were CPG or LSD carriers.

**Additional file 2: Figure S2.** Evaluation of *Galc* knockout in mouse pancreatic organoids.

**Additional file 3: Figure S3.** Increased proliferation in mouse *Kras*^*G12D*^*/Galc* knockout pancreatic organoids.

**Additional file 4: Table S1.** Forty-two Lysosomal storage disorder-associated genes

**Additional file 5: Table S2.** List of antibodies and drugs used in the study

**Additional file 6: Table S3.** Pathogenic variants identified in the Korean PDAC and normal control.

## Data Availability

Sequencing BAM files were deposited in the Sequence Read Archive (SRA Bioproject Accession # PRJNA929903).

## Abbreviations

CFNC: Cancer-free normal control
CPG: Cancer-predisposing gene
CQ: Chloroquine
DEG: Differentially expressed gene
GALC: Galactosylceramidase
GO: Gene ontology
GSEA: Gene set enrichment analysis
KEGG: Kyoto Encyclopedia of Genes and Genomes
KO: Knockout
IC50: Half maximal inhibitory concentration
LSD: Lysosomal storage diseases
OS: Overall survival
PDAC: Pancreatic ductal adenocarcinoma
PDO: Patient-derived organoid
PPV: Putative pathogenic variant
PTV: Protein truncating variant
RNA-seq: RNA sequencing
VCF: Variant calling format
WES: Whole exome sequencing
