# Supplementary material for: Genetic assessment of pathogenic germline alterations in lysosomal genes among Asian patients with pancreatic ductal adenocarcinoma
Source: J Transl Med. 2023 Oct 17;21:730. doi: 10.1186/s12967-023-04549-x (PMC10580633; doi:10.1186/s12967-023-04549-x)
Supplement: Supplementary file 1 — Additional file 1: Figure S1. Onset age in PDAC patients who were CPG or LSD carriers. [file 12967_2023_4549_MOESM1_ESM.docx]

**Additional Information –Figures and Figure legends**

**Fig. S1. Onset age in PDAC patients who were CPG or LSD carriers**. Age at diagnosis was compared in CPG carriers (n = 15), LSD (n = 32) and non-carriers (n = 371). Two PDAC patients, who were carriers of both the CPG and LSD genes, were included in the CPG carrier patient group. Unpaired t-test was used for statistical analysis. *P** < 0.05. Dashed line in the scatter dot plot indicates median value. PDAC, pancreatic ductal adenocarcinoma; CPG, cancer predisposition genes (*CHEK2, BRCA2, COL7A1, BRCA1, ATM, KRAS, TP53*); LSD, lysosomal storage disease (*GALC, HEXB, NPC1, IDUA, PSAP, MAN2B1, GAA, ARSA, HEXA, SGSH, NAGLU, MCOLN1, HYAL1, GUSB, GNPTG*).
