# Supplementary material for: Genetic assessment of pathogenic germline alterations in lysosomal genes among Asian patients with pancreatic ductal adenocarcinoma
Source: J Transl Med. 2023 Oct 17;21:730. doi: 10.1186/s12967-023-04549-x (PMC10580633; doi:10.1186/s12967-023-04549-x)
Supplement: Supplementary file 2 — Additional file 2: Figure S2. Evaluation of Galc knockout in mouse pancreatic organoids. [file 12967_2023_4549_MOESM2_ESM.docx]

**
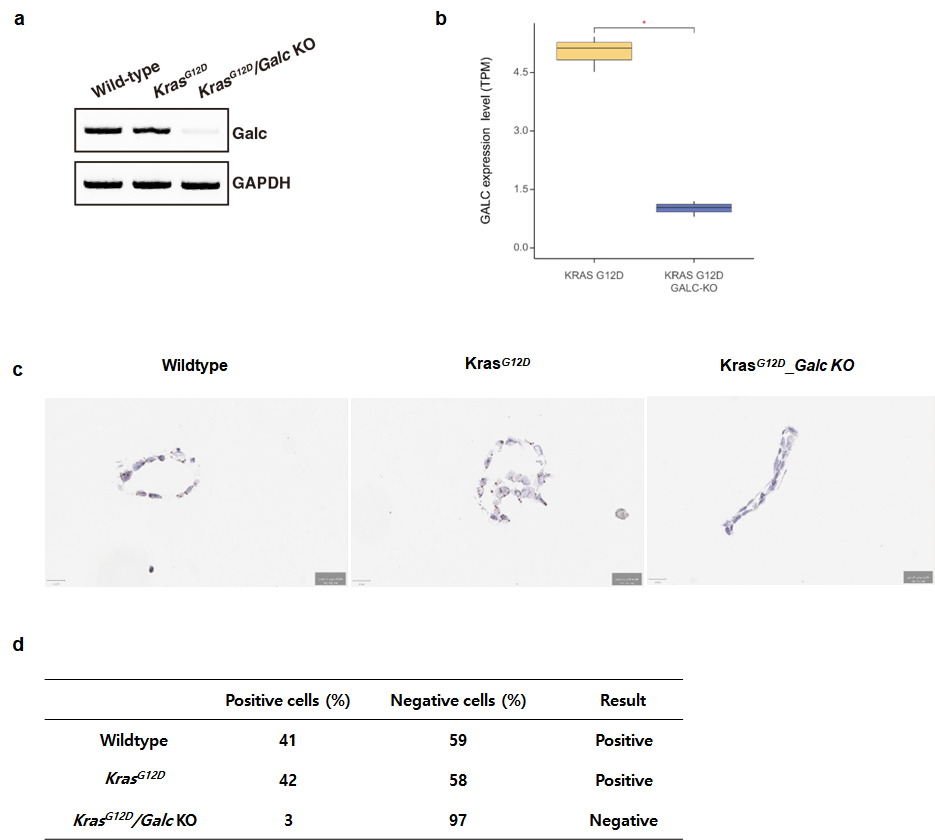
**

**Fig. S2. Evaluation of *Galc* knockout in mouse pancreatic organoids**. (a) The expression of GALC in mouse pancreatic wild-type, *Kras^G12D^*, and *Kras^G12D^/Galc* knockout organoids was examined using semi-quantitative RT-PCR. GAPDH was used as the internal control. The bands showed that Galc was efficiently knocked out in *Kras^G12D^/Galc* knockout organoids. (b) RNA sequencing was performed with mouse pancreatic wild-type, *Kras^G12D^*, and *Kras^G12D^/Galc* knockout organoids, and the expression level of GALC was accessed. The median expression level (TPM) of *Kras^G12D^/GalcKO* organoids was reduced to one-fifth of that of *Kras^G12D^* organoids. (c, d) Mouse pancreatic organoids were subjected to *in situ* hybridization with Galc probe. *Kras^G12D^/GalcKO* organoids showed the lowest percentage of Galc-positive cells. The number of Galc-negative and -positive cells was counted using the QuPath image analyzer and the percentage of Galc-negative and –positive cells are represented in table (WT: n = 262; *Kras^G12D^*: n = 91; *Kras^G12D^/Galc KO*: n = 65).
