# Supplementary material for: Genetic assessment of pathogenic germline alterations in lysosomal genes among Asian patients with pancreatic ductal adenocarcinoma
Source: J Transl Med. 2023 Oct 17;21:730. doi: 10.1186/s12967-023-04549-x (PMC10580633; doi:10.1186/s12967-023-04549-x)
Supplement: Supplementary file 3 — Additional file 3: Figure S3. Increased proliferation in mouse KrasG12D/Galc knockout pancreatic organoids. [file 12967_2023_4549_MOESM3_ESM.docx]

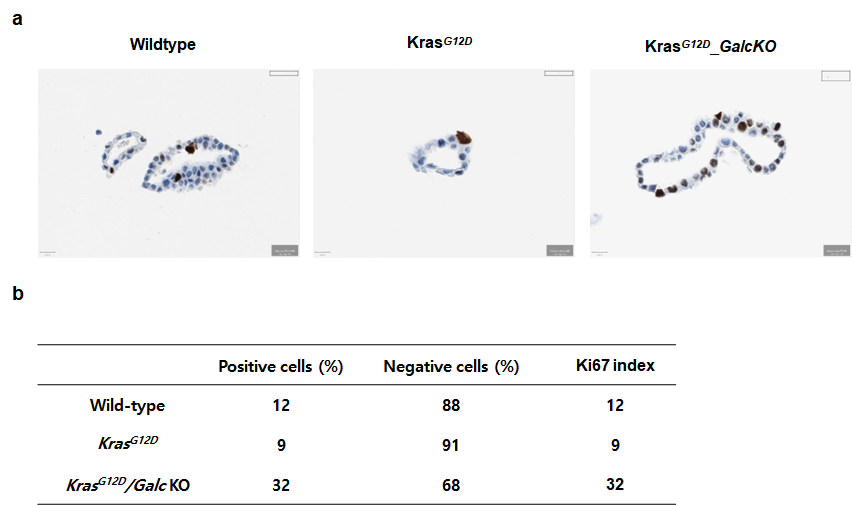


**Fig. S3. Increased proliferation in mouse *Kras^G12D^/Galc* knockout pancreatic organoids**. (a, b) Wild-type, *Kras^G12D^*, and *Kras^G12D^/Galc* knockout organoids were subjected to immunohistochemistry using the Ki-67 index. The percentage of Ki-67-positive cells was the highest in *Kras^G12D^/Galc* knockout organoids compared to that in the wild-type and *Kras^G12D^* organoids, which is consistent with the results of our immunofluorescence assay and RNA sequencing. The number of Ki67-negative and -positive cells was counted using theQuPath image analyzer and the percentage of Ki-67-negative and -positive cells are represented in table (WT: n = 527; *Kras^G12D^*: n = 198; *Kras^G12D^/Galc KO*: n = 202).
